# Systematic review of prevalence of pain among people with dementia living in the community

**DOI:** 10.1186/s12883-026-05071-5

**Published:** 2026-06-25

**Authors:** Anne-S. Helvik, Büşra Nur Temür, Sverre Bergh, Kjerstin Tevik

**Affiliations:** 1 Department of Public Health and Nursing, Faculty of Medicine and Health Sciences, Norwegian University of Science and Technology (NTNU), Trondheim, Norway; 2 Norwegian National Centre for Ageing and Health, Vestfold Hospital Trust, Tønsberg, Norway; 3 Department of Surgical Nursing, Faculty of Nursing, Akdeniz University, Antalya, Türkiye; 4 Research Center for Age-Related Functional Decline and Disease, Innlandet Hospital Trust, Ottestad, Norway

**Keywords:** Any pain, Behavioral assessment, Caregiver, Care worker, Community-living, Cognitive impairment, Daily pain, Multidimensional behavioral-observational assessment, Presence of pain, Proportion

## Abstract

**Background:**

Pain is common among people living with dementia (PLWD) in the community and is associated with substantial negative consequences for both individuals and caregivers; however, knowledge regarding its prevalence and assessment in a community living population with dementia remains limited. The aim of this systematic review was to examine the prevalence of pain and the pain assessment inventories used among PLWD at home.

**Method:**

This systematic review was registered in PROSPERO (CRD420251136436) and was conducted in accordance with the PRISMA 2020 statement. The searched databases include PubMed, MEDLINE, CINAHL, APA PsycInfo, AgeLine, the Cochrane Library, and Idunn, covering articles published from January 2000 to February 2026. Quantitative observational studies that reported pain through self-report questionnaires, staff- and/or caregiver- assessments were included to define the prevalence of pain in samples or subsamples of PLWD at home. The database search identified 1,296 records, of which 25 articles from 22 studies were included in the final review.

**Results:**

Sample sizes ranged from 36 to 1,379 PLWD at home. Pain was reported as any pain occurring within a defined timeframe, pain present on the day of assessment, pain meeting predefined severity criteria, or pain interfering with daily activities. The prevalence of pain among PLWD at home was consistently high, with higher prevalence estimates reported in studies assessing any pain compared with those applying severity or consequence-based criteria. The prevalence of any pain during the past month was found to vary between 36 and 76%. Considerable methodological heterogeneity was observed in terms of pain definitions, assessment methods, and inclusion criteria, which makes comparisons across studies difficult.

**Conclusion:**

The overall high prevalence of pain identified in this review underscores the need for systematic and standardized pain assessment for PLWD at home.

**Supplementary Information:**

The online version contains supplementary material available at https://doi.org/10.1186/s12883-026-05071-5.

## Introduction

The estimated global prevalence of dementia in 2019 was 57 million people, but the prevalence in 2050 is estimated to reach 150 million people [1], which is partly explained by demographic changes and longevity [2, 3]. More than 80% of those with dementia live in the community [4].

Pain is a common symptom among older adults with dementia. Internationally, studies have found the prevalence of pain to be up to 87.5% in people living with dementia (PLWD) at home [5]. However, the prevalence reported in studies of PLWD at home varies considerably [6–12]. The prevalence in studies of nursing home (NH) residents with dementia has also varied significantly [13–20]. Recent systematic reviews have evaluated the pain assessments used and summarized the pain prevalence among NH residents with dementia [21, 22], contributing to insight in assessment procedures and prevalence of pain among NH residents with dementia. To our knowledge, no systematic reviews have examined international published studies on the prevalence of pain and the assessment inventories used in PLWD at home [23, 24]. Consequently, there is a lack of knowledge regarding pain as a phenomenon in this group.

Pain among PLWD is often linked to medical conditions, such as arthritis, osteoporotic fractures, infections, injuries from falls, and gastrointestinal, cardiac and neuropathic pain [25–28]. Ethnicity is another characteristic that may be linked to the prevalence of pain [9, 12]. A study have reported that community living Latin-Americans with dementia were more likely to have pain than community living White-Americans with dementia [9], while another study reported that Chinese community living older adults with dementia had lower prevalence of pain than community living Latin-American with dementia [12]. However, no firm explanations were given for these findings. Furthermore, the experience of pain may be affected by neuropathological changes in the brain due to dementia that has its origins in white matter lesions and grey matter atrophy [29–31]. Recent evidence also suggests that alterations in brain regions involved in pain modulation, such as the periaqueductal gray (PAG), may contribute to altered pain perception and increased pain unpleasantness in people with Alzheimer’s disease [32]. Thus, it is expected that people living with severe dementia have pain more often than those with mild dementia, also among PLWD at home.

Pain is found to negatively affect mental health [27, 33], physical functioning [27], sleep [34], the ability to perform activities [35], and overall quality of life (QoL) [36]. Furthermore, pain among PLWD at home also impacts negatively on the caregiver – i.e., it is found to increase the caregiver burden [37]. Pain among persons with dementia is found to be associated with the presence of several neuropsychiatric symptoms [38], and the evidence for such a relation has been found to be strong between pain and agitation and/or aggression [27, 38].

Pain is an unpleasant sensation experienced by the individual, and self-reporting of pain is thus usually looked upon as the gold standard for assessing pain and is seen as a desirable goal to secure person-centered care [39]. However, dementia can complicate the self-reporting of pain, as the dementia impairs the memory and reduces the ability of the PLWD to verbally communicate pain [31]. People living with moderate to severe dementia might not reliably answer questions regarding pain [17]. Thus, some observational studies reporting the prevalence of pain invite the caregivers to respond on behalf of PLWD, either in addition to the PLWD’s responses or as a substitute for self-reporting pain. Pain assessments used in prevalence studies could comprise one or a few single questions about pain [40] or multidimensional behavioral-observational assessment inventories of pain [7] completed by paid care workers or voluntary caregivers, such as the Mobilization-Observation-Behaviour-Intensity-Dementia-2 (MOBID-2) Pain Scale [41], the Pain Assessment in Advanced Dementia (PAINAD) Scale [42], Pain Assessment in individuals with Impaired Cognition (PAIC15) [43] or others. Some studies may collect information about the prevalence of documented pain in medical or clinical records/charts, diagnoses commonly associated with pain, and/or the use of analgesics [28, 44, 45] as proxy for assessments of the prevalence of pain reported by the PLWD, caregivers, or care workers. Such substitute assessments may have very limited accuracy – for example, pain among PLWD at home may not be reported in medical records or charts if they are underdiagnosed or undertreated [46, 47], or if they are without pain due to treatment with analgesics. The pain assessment procedures and inventories for persons with dementia have improved since 2000 [48] and thus deemed more valid and relevant for clinical practices than older studies. In studies of persons with dementia, pain may be assessed as present pain, persistent pain, chronic pain, and/or intermittent pain [21]. Studies of the prevalence of present pain among PLWD at home may report the presence within a timeframe [49], at the assessment day [40], of a certain severity [50] and/or as having consequences for life [6]. A systematic review may contribute to providing an overview of the methods used to assess pain among the PLWD at home.

It is reported that PLWD at home receive pain treatment (over-the-counter medication and prescribed medications) less frequently than their peers without dementia [46]. A systematic review of the prevalence of pain among PLWD at home can facilitate pain assessment in clinical practice as well as the non-pharmacological and pharmacological treatment that may improve their everyday life. In addition to healthcare staff, a systematic review of observational studies will provide relevant information for policymakers and healthcare service management personnel [51]. Therefore, we aimed to systematically review published observational studies reporting the prevalence of pain among PLWD at home published from 2000 to February 2026, irrespective of whether pain was assessed through self-report, caregiver report, or care worker report, and independent of the assessment method, sample size, or statistics used.

## Materials and methods

The PRISMA 2020 statement [52] guided this systematic review. A PRISMA checklist is available in Supplementary Table 1 (S1). The systematic review is registered in PROSPERO (CRD420251136436), no protocol is published.

### Search strategy and study selection

In this review, the authors consulted the academic librarian service at the Norwegian National Centre for Ageing and Health, and they designed, discussed, and conducted a systematic, computerized search in the PubMed, MEDLINE, CINAHL, APA PsycInfo, AgeLine, Cochrane Library, and Idunn databases for articles published from January 2000 to February 2026. The last search was performed on February 03, 2026. The following search terms were used in PubMed: (((("people living with dementia") OR (dementia[MeSH Terms])) OR (cognitive impairment[MeSH Terms])) AND (pain[MeSH Terms]) AND (2000:2025[pdat])) AND (prevalence) Filters: English. Detailed search strategies for each database are presented in Supplementary Table 2. (S2). The articles were exported to EndNote Version 21. In addition, the reference lists of the included articles were screened to find studies that were not detected in the systematic searches.

The study inclusion criteria were:

- The study was an observational study with a quantitative design (longitudinal or cross-sectional) or included baseline findings from a designed intervention study,
- Study participants with dementia who were community-living (living at home),
- Dementia was established based on medical diagnosis and/or defined assessments criteria,
- Pain was reported by use of self-report questionnaires, staff assessments, and/or caregiver assessment, and
- The study was published in a scientific peer-reviewed journal, was written in English and from 2000 or newer.

Studies were excluded from the review if they were:

- Overview articles, or review studies,
- Theoretical, qualitative, or editorial articles, or comments on studies,
- Determining dementia by using subjective reports from caregivers and/or self-report from individuals with dementia,
- Without sub-group analyses of PLWD at home,
- Using presence of pain as an inclusion or exclusion criterion for study participation,
- Using substitute measures for prevalence of pain, such as prescription of analgesics, chart reviews of documented pain, or diagnoses commonly associated with pain,
- Not reporting the prevalence of pain, or
- Reporting the prevalence of pain without documenting the findings.

### Identification of relevant studies

Each article’s title and abstract were screened by the first and last author (ASH & KT) to search for potential eligibility. The full-text versions of the articles were obtained if it was unclear whether the study met the inclusion criteria. Thereafter, two authors read all full-text articles, and any uncertainty regarding study eligibility was resolved through discussion between the same two authors (ASH & KT).

### Data extraction

The first author (ASH) extracted all relevant information from the eligible studies, including the year of publication, year of data collection, study country, study population/sample, study design, number of participants, age and sex of participants, inclusion criteria, description of dementia, how pain was assessed, and the timepoint and timeframe for the assessments. The last author (KT) reviewed this information. A list with descriptions of the original articles was developed for this review, including their aims, sample characteristics, and findings related to our study.

### Critical appraisal

The critical appraisal checklist from Joanna Briggs Institute (JBI) [53] for prevalences studies was used together with predefined criteria regarding ethical approval [54, 55]. The same two authors (ASH & KT) critically appraised all articles independently, and disagreement was resolved by discussion between these two authors. Quality assessments were not used as a basis for excluding studies but to gain a better understanding of the overall quality of the literature in the field. A score of 1 was given for + (plus, criterion present), and a score of 0 was given for both – (minus, criterion absent) and ? (question mark, unclear if criterion was present). The sum score of the quality assessment of each study could vary between 0 and 10, where 10 points indicated fulfilling the criteria by 100%.

### Ethics

No ethical approval was required because the study used secondary data.

## Results

PRISMA 2020 flow-diagram depicting the records that were identified, screened, assessed for eligibility, and the full-text articles reviewed and included in this review.

### Literature search and selection

**Fig. 1:**
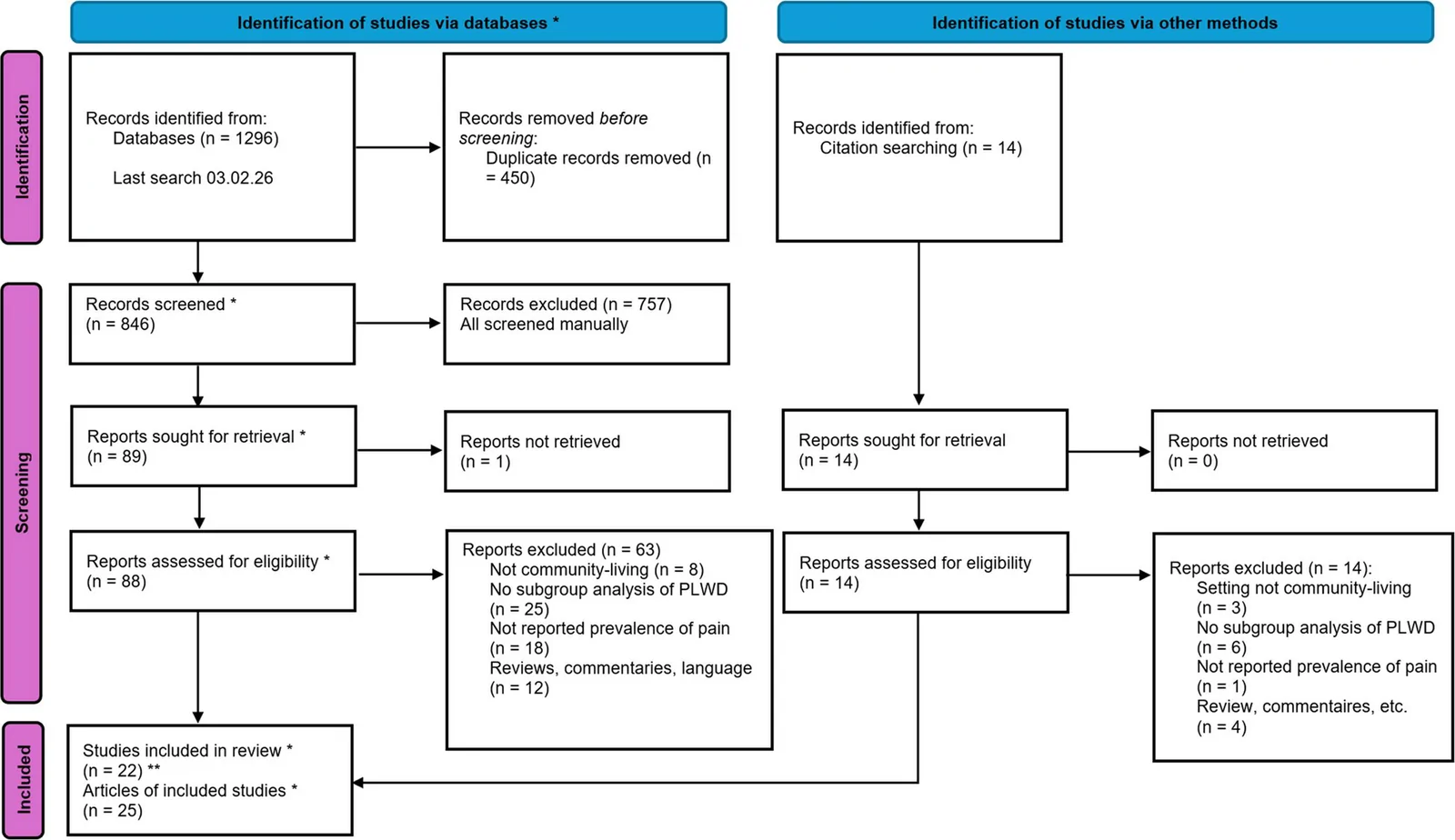
PRISMA 2020 flow diagram. Search of databases and citations for systematic review. *Read and assessed by first and last author (ASH & KT). **One study has presented prevalence of pain in three articles and another in two articles. Thus, the total number of articles included in the review is 25 and the number of studies is 22. Source: Page ML, et al. BMJ 2021;372:n71. https://doi.org/10.1136/nmj.n71. Licensed under CC BY 4.0

The database search identified 1,296 records. After duplicates were removed (450 records), 846 records remained to be screened manually (title and abstract by ASH & KT), of which 89 full-text records were considered for inclusion (by ASH & KT). Of these, 25 articles were included, coming from 22 studies. In addition to articles found through the systematic search, we identified 14 new records in the reference lists that were potentially of interest. All of those were retrieved, but none of them fulfilled the inclusion criteria. The final number of articles included was therefore 25. Figure 1 presents the PRISMA 2020 flow diagram [52], and provides an overview of the search, information about articles that were identified, screened, and assessed for eligibility, and the articles included in the review.

#### Settings and samples

Table 1 presents the characteristics of the included articles (*n* = 25) and the assessments used to explore pain in community-living studies including PLWD. One small study (*n* = 115) was presented in three articles [47, 56, 57] and another small one (*n* = 88) in two articles [36, 58]. The sample sizes of individual studies ranged from 36 [59] to 1,379 [12] PLWD at home. The age of the participants with dementia ranged from median 53 years [59] years to mean age 86 years [7, 10]. All studies included both men and women. In total, nine of the 22 studies (nine of the 25 articles) included participants with and without dementia. One study included both residents with and without dementia according to care setting (community-living and NH) [50]. In total, 11 studies (14 articles) were conducted in North America, six in Europe, two in Latin America (Brazil and Colombia), two in Asia (Taiwan and China), and one in several low and middle-income countries (including seven countries).

**Table 1:** Study characteristics of articles reporting prevalence of pain among People Living With Dementia (PLWD) at home

	**Author, year, location, design**	**Aim relevant for this review**	**Sample of PLWD**	**Dementia/cognition**	**Assessment of pain &** reported by whom	**Findings &** prevalence of pain among PLWD
1	Barry et al. 2016 [40] Northern Ireland, UK CS	To explore pain and factors associated with pain among PLWD	*n* = 75 Mean age (SD): 79.1 (7.7) years Female: 53.3%	Diagnosed at a memory clinic 92% with mild to moderate dementia Mean (SD) MMSE 17.8 (6.8)	Any pain just now and Any pain on an average day using 7-point VDS Reported by PLWD and Caregiver	On an average day: 57.3% had self-reported pain 70.7% had caregiver-reported pain Pain just now: 36.0% had self-reported pain 53.3% had caregiver-reported pain
2	Bentur et al. 2021 [7] Israel CS	To compare identification of pain among people living with advanced stage dementia assessed by family and paid care workers	*n* = 82 Mean age (SD): 86.4 (7.0) years Female: 52.5%	Diagnosed with advanced dementia No further cognitive information was reported	Pain according to PAINE > 2.75 PAINAD > 2 General impression of pain past 2 weeks, One item, (PPI > 3) Reported by Care workers (paid) and Caregiver	Having pain using PAINE 46.3% had care worker-reported pain 37.8% had caregiver-reported pain Having pain using PAINAD 46.3% had care worker-reported pain 51.2% had caregiver-reported pain General impression of pain 12.7% had care worker-reported pain 16.0% had caregiver-reported pain
3	Bhattacharjee et al. 2017 [60] USA CS	To explore pain and other factors for depression treatment among depressed PLWD	*n* = 173 ≥ 75 years: 78.7% Female: 69.7%	Diagnosed with ICD-9-CM code or used cholinesterase inhibitors/memantine No further cognitive information was reported	Any pain Pain interfering with normal work outside the home or housework, past 4 weeks Not said who reported pain (PLWD/caregiver)	Some form of pain 79.1% had pain (not specified which item used/merged)
4	Duan et al. 2024 [8] China CS	To explore pain and the association between pain severity and presence of dementia	Total *n* = 4559 PLWD *n* = 1380 Mean (SD) age: 72.1 (5.8) years Female: 56.9%	Dementia diagnoses were established by MMSE cutoff, which adjusted for education No further cognitive information was reported	Being troubled by any bodily pain (Yes/No) (no timeframe) Reported by PLWD	Troubled by bodily pain 63.1% had self-reported pain
5	Gitlin et al. 2014 [58] USA CS	To explore pain and other modifiable risk factors for poor QoL among PLWD	*n* = 88 plus their caregivers PLWD Mean (SD) age: 81.7 (8.0) years Female: 52.3%	Established dementia with MMSE ≥ 10 Mean (SD) MMSE: 17.7 (4.6)	VAS with Faces Pain Scale (0–10) (≥ 1 = pain) Reported by PLWD	Any pain present 42.5% had self-reported pain
6	Harrison et al. 2019 [50] USA CS	To explore pain and other conditions among moderately severe PLWD by care setting (community-living or institution)	Total *n* = 728 PLWD at home *n* = 4 99 Mean age (SD): 82.1 (8.2) years Female: 59.8%	Incident of moderately severe dementia when meeting the NHATS criteria and appearing between 2012 and 2016 in the study No further cognitive information was reported	Pain according to NHATS: Bothersome pain and Pain-limiting activities over the past month Reported by PLWD or Caregiver (50.3% were caregiver-reported among community-living participants)	Bothersome pain in past month 70.8% had such pain Pain limiting activities in past month 52.7% had such pain
7	Hayes-Larson et al. 2021 [9] USA CS	To explore pain and other conditions by racial/ethnical disparities among older adults with and without dementia	Total *n* = 10,886 Age ≥ 75 years: 58.1% Female: 58% PLWD *n* = 1304 Mean (SD) age: ? years Female:?%	Dementia according to NHATS criteria No further cognitive information was reported	Pain according to NHATS Bothersome pain during the past month Reported by PLWD or Caregiver (40% were caregiver-reported among those with dementia)	Predicted prevalence bothersome pain adjusted for gender and age 52% of Afro-Americans had such pain 67% of Latin-Americans had such pain 57% of Whites had such pain
8	Hodgson et al. 2014 [36] USA CS	To explore pain and other conditions and their importance for QoL among PLWD	*n* = 88 Mean (SD) age: 81.7 (8.0) years Female: 52.3% Same study as Gitlin et al., 2014	Mild to moderate dementia MMSE ≥ 10 Mean (SD) MMSE: 17.7 (4.6)	VAS with Faces Pain Scale (0–10) (≥ 1 = pain) Reported by PLWD	Any pain present 42.5% had self-reported pain
9	Hunt et al. 2015 [35] USA CS	To explore prevalence of pain and pain medication use among PLWD	*n* = 802 Aged ≥ 80 years: 67.2% Female: 65%	Using a modified algorithm from NHATS to define dementia (or not) Those with questionable dementia (not meeting all criteria set in algorithm) were excluded	Bothersome pain and activity-limiting pain during the past month Reported by PLWD or Caregiver (49.6% were caregiver-reported)	Bothersome pain in past month 63.5% had such pain Pain limiting activity in past month 43.3% had such pain
10	Haasum et al. 2011 [10] Sweden CS	To compare pain and pain treatment by place of living among older adults with and without dementia	Total *n* = 2610 PLWD at home *n* = 119 Mean age (SD): 86.7 (6.5) years Female: 79.0%	Diagnosis according to DSM-IV criteria Mean (SD) MMSE: 19.5 (4.8) in PLWD at home	Any pain in past 4 weeks Reported by PLWD	Any pain in past 4 weeks 36.1% had self-reported pain
11	Jensen-Dahm et al. 2012 [61] Denmark RCT baseline	To explore self- and proxy-reported pain among PLWD	*n* = 321 plus their caregivers PLWD Mean (SD) age: 76.2 (7.1) years Female: 54.8%	Diagnosis according to DSM-IV criteria Alzheimer’s disease, mild stage, MMS ≥ 20 Mean (SD) MMSE: 24 (2.6)	Pain question in EQ-5D Dichotomized pain/discomfort problems (no vs some and extreme) actual day Reported by PLWD and Caregiver	Having pain/discomfort problem actual day 32.9% had self-reported pain 52.0% had caregiver-reported pain (= primary caregiver)
12	Kroenke et al. 2022 [49] USA RCT baseline	To explore pain and other symptoms among PLWD of advanced stage	*n* = 201 plus their caregivers PLWD Mean (SD) age: 83.1. (8.0) years Female: 67.7%	Diagnosed with dementia Moderate to severe stage of dementia (FAST-stage 5–7) 88.1% had severe stage (FAST 6–7)	Pain was assessed using SM-EOLD Reporting: Any pain in past 90 days Never Once a month 2/3 days a month Once a week Several days a week Every day And Summing up: Pain at least weekly Reported by Caregiver	Any pain in the past 90 days 79.1% had pain once or more in past 90 days More frequently reported pain 9.5% had pain once a month 13.9% had pain 2/3 days a month 10.9% had pain once a week 14.9% had pain several days a week 29.9% had pain every day 55.7% had pain at least weekly
13	Krulewitch et al. 2000 [5] USA CS	To compare PLWD-reported and caregiver-reported pain among PLWD	*n* = 156 plus their caregivers PLWD Age range: 65–98 years Female: 83%	Dementia was recorded in charts and MMSE confirmed the likelihood of dementia Mean (SD) MMSE:15.7 (5.9) Mean (SD) FAST score: 6.2 (0.8)	Pain was assessed by three inventories: 1. Faces Pain Scale (0–6) with faces showing no pain to excruciating pain 2. VAS scale, a line from 0 (no pain) to 100 (worst pain) 3. PIS 5 items (score 5–25) Reported by PLWD and Caregiver	Any pain 87.5% had self-reported pain (n = 104 completing one of the pain scales)
14	Li et al. 2020 [11] Taiwan CS	To explore frailty, pain, and other health problems among people with and without dementia	Total *n* = 2693 All 65 years and older PLWD a) *n* = 365 with no frailty Age ≥ 75 years: 46.5% Female: 55.4% b) *n* = 330 with frailty Age ≥ 75 years: 65.0% Female: 70.5%	Cognitive measure (MMSE) for probably dementia No further cognitive information was reported	Pain question in EQ-5D Dichotomized pain/discomfort problems (no vs some and extreme) actual day Reported by Participant	Reporting pain/discomfort problem actual day 16.5% of those with no frailty 51.2% of those with frailty
15	Lopes et al. 2016 [62] Brazil CS	To explore pain and other conditions among older adults with and without dementia	Total *n* = 1705 Mean (SD) age: 70.6 (8.0) years Female: 63.9% PLWD *n* = 325 Age ≥ 75 years: 54.5% Female: 72%	Cognitive (MMSE) and functional impairment as measure for dementia No further cognitive information was reported	Any pain on most days Diffuse pain (pain in several body parts during past month) Reported by Participant	Any pain most days 56.6% had such pain Diffuse pain in past month 38.8% had such pain
16	Mäntyselka et al. 2004 [6] Finland CS	To explore pain among older adults with and without dementia	*n* = 523 All 75 years and older PLWD *n* = 75 Mean (SD) age: 83 (?) years Female: 73.3%	Diagnosed with DSM-IV and DSM-III-R criteria Numbers with Mild dementia: 43 Moderate dementia: 27 Severe dementia: 5	Pain reported as: Any pain in past month Any daily pain Pain every day interfering with routine activities Daily pain at rest Reported by Participant (in PLWD: n = 64) or Caregiver	Any pain 43% had such pain in past month 23% had such pain daily Pain interfering with routine activities daily 19% had such pain Daily pain at rest 4% had such pain
17	Orgeta et al. 2015 [63] UK CS	To explore pain and factors associated with pain among PLWD	*n* = 488 plus their caregivers PLWD Mean (range) age: 75.6 (54–95) years Female: 49.6%	Diagnosed with dementia according to DSM-IV criteria for any dementia Degree of dementia: mild to moderate No further cognitive information was reported	Pain question in EQ-5D Dichotomized pain/discomfort problems (no vs some and extreme) actual day Reported by PLWD and Caregiver	Pain/discomfort problems actual day: 45.3% had self-reported pain 59.2% had caregiver-reported pain Agreement between PLWD-reported and caregiver-reported pain (Yes/No) was 65% (kappa = 0.25)
18	Prince et al. 2011 [12] India China Latin America CS	To explore pain and other conditions among people with and without dementia in low and middle-income countries	Total *n* = 15,022 Mean (SD) age varies between 71.3 (6.1) and 75.3 (7.5) years in state and region Female: 61.6% PLWD *n* = 183 India *n* = 140 China *n* = 1056 Five Latin American countries No information in subsamples of PLWD about age and gender distribution	Dementia was diagnosed with DSM-IV criteria or by research criteria (from the 10/66 study) No further cognitive information was reported	Modified version of McGill Pain Scale Assessing intensity and extent of pain at present, reporting any pain Reported by Participant	India 43.2% with mild dementia had any pain present 11.1% with moderate or severe dementia had any pain present China 16.1% with mild dementia had any pain present 7.0% with moderate or severe dementia had any pain present Latin America (5 countries) 30.1% with mild dementia had any pain present 17.9% with moderate or severe dementia had any pain present
19	Regier et al. 2018 [64] USA RCT baseline	To explore pain and other factors associated with restlessness among PLWD with one or more NPS	*n* = 569 plus their caregivers PLWD Mean (SD) age: 82.3 (8.7) years Female: 60.8%	Diagnosed by a physician with moderate dementia according to MMSE cutoff (≤ 23) Mean (SD) MMSE:12.9 (8.1)	A 5-point Likert scale of degree of pain (no pain to extreme pain) experienced by PLWD Any pain (Yes/No) a) Over the past few weeks b) At present time c) How much pain interferes with daily activities Reported by Caregiver	Any pain over the past few weeks 75.8% of all respondents had pain 79.6% with restlessness had pain 68.8% without restlessness had pain Prevalence of any present pain and pain interfering with daily activities was not reported
20	Shega et al. 2004 [56] USA CS	To explore pain among PLWD and the agreement between their and caregiver rating	*n* = 115 plus their caregivers PLWD Mean (SD) age: 84.1 (6.7) years Female: 75.7%	Clinically diagnosed at out-patient clinic Mean (SD) MMSE: 16.6 (7.2)	VDS Presence and severity of pain on a 7-point response scale Item: How much pain are you (PLWD) experiencing just now? Reported by PLWD and Caregiver	Any pain just now 32% had self-reported pain 52% had caregiver-reported pain Pain just now being ≥ moderate 11% had self-reported pain 25% had caregiver-reported pain
21	Shega et al. 2005 [57] USA CS	To explore pain and factors associated with pain rated by PLWD and caregivers	*n* = 115 plus their caregivers PLWD Mean (SD) age: 84.1 (6.7) years Female: 75.7% Same sample as Shega et al., 2004	Clinically diagnosed at outpatient clinic Mean (SD) MMSE: 16.6 (7.2)	VDS Presence and severity of pain on a 7-point response scale Item: How much pain are you (PLWD) experiencing just now? Reported by PLWD and Caregiver	Any pain just now 32% had self-reported pain 53% had caregiver-reported pain
22	Shega et al. 2006 [47] USA CS	To explore pain and pain treatment among PLWD without cancer	*n* = 115 plus their caregivers PLWD Mean (SD) age: 84.1 (6.7) years Female 75.7% Same sample as Shega et al., 2004	Clinically diagnosed at out-patient clinic Mean (SD) MMSE: 16.6 (7.2)	VDS pain item Presence and severity of pain on a 7-point response scale Item: How much pain do you (PLWD) have on an average day? Reported by PLWD	Pain on an average day 54% had self-reported pain
23	Tabares-Villamizar et al. 2025 [59] Colombia CS	To explore pain and most frequent symptoms among people with early-onset Alzheimer’s disease and their care needs	*n* = 36 Median (IQR) age: 53 (8) years Female: 63.9%	Diagnosed with early-onset Alzheimer’s disease with E280 A genetic variant of PSEN1 11.1% moderately severe dementia 88.9% severe and very severe dementia Screened with GDS/FAST	Pain according to PAINAD > 0 and ESAS-r, 1 item > 0 Presence of any pain Reported by Caregiver	Any pain at assessment 52.8% had pain according to PAINAD > 0 44.4% had pain according to ESAS-r > 0
24	Wang et al. 2018 [65] USA CS Pilot study	To explore pain interfering with activities among physically healthy PLWD (not taking daily analgesics)	*n* = 52 Mean (SD) age: 78.2 (7.9) years Female: 55.8%	Diagnosis confirmed by GP Mean (SD) MMSE: 18.5 (5.1)	BPI-SF (9-item self-report) reporting severity (in levels, no pain to high pain) of pain today and average pain Pain interfering with daily activities (7 activities) Reported by PLWD	Any pain 23.1% had pain on enrollment day Some pain 13.5% had some pain today 28.8% had some pain on average Pain interfering with activities 21.2% had such pain
25	Wang et al. 2022 [46] USA CS	To explore pain and pain treatment among older adults with and without dementia	Total *n* = 16,380 PLWD *n* = 734 Mean (SD) age: 76.5 (11.7) years Female: 58.9%	Using the score from the Telephone Interview for Cognitive Status Categorizing results in person with: Dementia No dementia or CI No dementia but some CI	Troubled by pain (often being troubled with pain) (Yes/No) Pain severity (degree of pain most of the time: 0–4) Pain interference (pain making it difficult to do usual activities) (Yes/No) One item for each type of pain assessment Reported by PLWD or Caregiver	Troubled by pain often 46.2% had such pain often Pain most of the time 12.5% had mild pain 20.0% had moderate pain 13.5% had severe pain Pain interfering with usual activities 33.4% had such pain

*BPI-SF* Brief Pain Inventory – Short Form, *CI* Cognitive Impairment, *CS* Cross-Sectional Study, *DSM-III-R* Diagnostic and Statistical Manual of Mental Disorders (3rd edition), *Revised; DSM-IV* Diagnostics and Statistical Manual of Mental Disorders (4th edition), *ESAS-r* Edmonton Symptom Assessment Questionnaire, *EQ-5D* EuroQol 5 Dimensions, *FAST* Functional Assessment Staging Tool, *ICD-9-CM* International Classification of Diseases (9th revision), Clinical Modification, *GDS* Global Deterioration Scale, *GP* General Practitioner, *MMSE* Mini Mental State Examination Score, *IQR* Interquartile Range, *NHATS* National Aging and Trends Study, *NPS* Neuropsychiatric Symptoms, *PAINAD* Pain Assessment in Advanced Dementia, *PAINE* Pain Assessment in Non-Communicative Elderly Tool, *PPI* Present Pain Indicator, *PIS* Pain Intensity Scale, *PLWD* Person Living with Dementia, *QoL* Quality of Life, *RCT* Randomized Clinical Trial, *SD* Standard Deviation, *SM-EOLD* Symptom Management in End-of-Life Dementia Scale with One Pain Item, *UK* United Kingdom, *VAS* Visual Analog Scale, *VDS* Verbal Descriptor Scale

### Design

Of the 22 studies, three reported baseline results from intervention studies that did not recruit or exclude participants on the basis of pain [49, 61, 64]. The other studies reported cross-sectional results.

### Quality assessment

Table 2 provides a description of the quality assessment of the studies included. Two articles received scores of ≥ 90%, indicating strong quality, eight articles had good quality (received 70–89% of the scores), nine articles had fair quality (received 50–69% of the scores), and six articles received < 50% of the scores (poor quality). Most of the studies with good quality used an appropriate sample to assess pain in community living people with dementia but failed to inform about the precision of the prevalence found of pain (95% confidence interval) as required by the JBI criteria require. The studies with poor quality had all described the study setting adequately, but they had a small sample size, poor response rate, lacked sufficient analysis of covered sample and did not fulfill the requirements for appropriate recruitment of participants in line with the JBI criteria.

**Table 2:** Critical appraisal

	**Article**	**1**	**2**	**3**	**4**	**5**	**6**	**7**	**8**	**9**	**10**	**Sum score**
1	Barry et al., 2016 [40]	+	-	-	+	-	+	-	-	-	+	4
2	Bentur et al., 2021 [7]	+	-	-	+	-	+	+	-	-	+	5
3	Bhattacharjee et al., 2017 [60]	+	+	-	+	-	?	-	-	-	-	3
4	Duan et al., 2024 [8]	+	+	+	+	+	?	-	-	-	+	6
5	Gitlin et al., 2014 [58]	?	-	-	+	-	+	+	-	-	+	4
6	Harrison et al., 2019 [50]	+	+	+	+	+	+	-	-	-	+	7
7	Hayes-Larson et al., 2021 [9]	+	+	+	+	?	+	-	+	-	+	7
8	Hodgson et al., 2014 [36]	?	-	-	+	-	+	+	-	-	+	4
9	Hunt et al., 2015 [35]	+	+	+	+	+	+	+	+	+	+	10
10	Haasum et al., 2011 [10]	+	+	–	+	+	?	-	-	+	+	6
11	Jensen-Dahm et al., 2012 [61]	+	-	+	+	-	+	-	-	-	+	5
12	Kroenke et al., 2022 [49]	+	-	+	+	-	+	-	-	-	+	5
13	Krulewitch et al., 2000 [5]	+	+	-	+	+	+	-	-	+	+	7
14	Li et al., 2020 [11]	+	+	+	+	+	+	-	-	+	+	8
15	Lopes et al., 2016 [62]	+	+	+	+	+	?	+	-	+	+	8
16	Mäntyselka et al., 2004 [6]	+	+	-	+	+	?	+	-	+	+	7
17	Orgeta et al., 2015 [63]	+	-	+	+	-	+	+	-	-	+	6
18	Prince et al., 2011 [12]	+	+	+	+	+	+	+	-	+	+	9
19	Regier et al.,2018 [64]	-	-	+	+	-	?	?	-	-	+	3
20	Shega et al., 2004 [56]	+	-	-	+	+	+	+	-	+	+	7
21	Shega et al., 2005 [57]	+	-	-	+	?	+	+	-	?	+	5
22	Shega et al., 2006 [47]	+	-	-	+	+	+	+	-	+	+	7
23	Tabares-Villamizar et al., 2025 [59]	+	-	-	+	+	+	+	-	-	+	6
24	Wang et al., 2018 [65]	-	-	-	+	-	+	-	-	-	+	3
25	Wang et al., 2022 [46]	+	+	+	+	-	?	+	-	-	+	6

Using JBI criteria for prevalence studies (items 1–9) and one item regarding ethical handling:

1) Was the sample frame appropriate to address the target population? 2) Were study participants recruited in an appropriate way? 3) Was the sample size adequate? 4) Were the study subjects and setting described in detail? 5) Was data analysis conducted with sufficient coverage of the identified sample? 6) Were valid methods used for the identification of the condition? 7) Was the condition measured in a standard, reliable way for all participants? 8) Was there appropriate statistical analysis? 9) Was the response rate adequate, and if not, was the low response rate managed appropriately? 10) Had the study received ethical approval?

### Assessment of pain

Pain was assessed systematically in all included studies. In nine articles, pain was self-reported [8, 10, 11, 36, 47, 58, 62, 63, 65], whilst in four articles the participants’ pain was reported by their caregivers [40, 49, 59, 64]. Additional, five articles included both self-reported and caregiver-reported pain information [5, 40, 56, 57, 61], and five articles used either self-reported or caregiver-reported information [6, 9, 36, 46, 50], depending on the severity of dementia and the participants’ ability to communicate. In one study, pain was assessed both by family caregivers and care workers [7], and one article did not clarify who reported the pain [60]. Only two small studies used observational multidimensional pain assessment inventories [7, 59], whilst the other studies used one or several single pain items to assess pain independent of who responded to the items. PLWD receiving pain treatment but not experiencing pain at the assessment timepoint were categorized as not having pain. The presence and severity of pain were reported.

The included studies explored the presence of any pain within a defined timeframe (from past 90 days to daily at rest) (reported in 7 articles with 11 definitions), any present pain (reported in 13 articles and assessed with 11 definitions), presence of pain defined by certain severity criteria such as troublesome, mild or higher, moderate, clinically relevant, etc. (reported in 9 articles and addressed with 13 definitions), and presence of pain interfering with accomplishing tasks such as work and activities (reported in 7 articles using 6 definitions of tasks to be accomplished) (see Table 3). Any pain within a defined timeframe was most often assessed over the past month (including a few weeks and four weeks) and was used in three studies [6, 10, 64]. Present pain was most often assessed using the EQ-5D (EuroQol 5 Dimensions), 1-pain item [11, 61, 63], and VDS (Verbal Descriptor Scale), 1-pain item [9, 35, 50], which were used in three studies each. The most common question regarding experiencing pain of a certain degree concerned having bothersome pain during the past month and was used in three studies [9, 35, 50]. None of the studies reported pain related to movements in general.

**Table 3:** Categories of pain

Category of pain	Number of articles*	**References**
Any pain within a timeframe	7	[6, 10, 40, 47, 49, 62, 64]
Any present pain at assessment day	13	[5, 11, 12, 36, 40, 56–59, 61, 63–65]
Pain defined by some severity criteria	9	[7–9, 35, 46, 50, 56, 62, 65]
Pain interfering with accomplishing tasks	7	[6, 35, 46, 50, 60, 64, 65]

^*^Some articles report pain in more than one category

### Prevalence of pain

The prevalence of pain varied considerably among PLWD at home and differed considerably with category of pain explored. The highest prevalence of any pain within a timeframe was 79.1%, in one study that reported pain within the past 90 days and that was assessed by the caregiver [40]. The prevalence of any pain during the past month varied between 36.1% [10] and 75.8% [64]. The lowest prevalence of pain was reported in a study that assessed any daily pain at rest (4%) [6]. Only one study reported any daily pain at rest. The highest reported prevalence of any present pain was 87.5% [5] and the lowest prevalence was 23.1% [65].

The prevalence of pain fulfilling certain severity criteria decreased partly by increasing level of severity and frequency of pain required. The prevalence of pain being troublesome during the past month varied between 57.9% [9] and 70.8% [50], whilst the prevalence of having pain interfering with tasks such as work or activities varied between 19% [6] and 52.7% [50].

In a few studies, the prevalence of pain was explored by certain characteristics of PLWD [9, 11, 12, 64]. One study explored pain by frailty and found self-reported prevalence of pain higher among PLWD with frailty than without frailty [11]. Only one study reported a sub-group analysis of the prevalence of any pain according to the severity of dementia among community living adults in different parts of the world (India, China, and Latin America), showing that those with mild dementia self-reported any pain more often than those with moderate to severe dementia [12]. However, the prevalence of any pain varied considerably by country. Among those with mild dementia, the prevalence of any pain was 43.2% in India, 30.1% in Latin America, and 16.1% in China, compared to 11.1%, 17.9%, and 7.0%, respectively, among those with moderate to severe dementia.

The third study performing sub-group analysis explored prevalence of pain by ethnicity and found that age- and gender-adjusted prevalence of pain varied between Afro-American, Latin-American, and White American, and was highest among PLWD of Latin-American ethnicity, and lowest in PLWD of Afro-American ethnicity [9]. A final study explored the caregiver-reported prevalence of pain among PLWD with and without restlessness, and found pain to be more common among those with restlessness than those without [64].

Some of the variations of prevalence of pain in PLWD at home seem to be attributable to those who responded to the questions about pain among PLWD. In general, caregivers reported a higher prevalence of pain than the PLWD did in the same study, independent of the assessment method used [5, 40, 56, 57, 61]. Furthermore, the paid care workers reported a lower prevalence of pain than the family caregivers of PLWD [7].

## Discussion

In total, 25 articles (22 studies) published in international peer-reviewed journals in the period between 2000 and 2026 reported the prevalence of pain among PLWD at home by self-report, caregiver-report, and/or care worker-report. Pain was reported either as any pain occurring within a defined timeframe, any pain present at assessment day, pain fulfilling defined severity criteria, or pain interfering with the accomplishment of tasks. The prevalence of pain was considerably high in PLWD at home, but the prevalence was generally higher in studies of any pain (either within a timeframe or at assessment) than in studies that required certain criteria for severity or consequence. The methodology varied considerably, by definitions of pain, methods and assessments used to report pain, and inclusion criteria, which makes comparisons difficult.

In the present systematic review of pain among PLWD at home, the prevalence of pain varies partly due to how pain is defined. An exceptionally low prevalence (4%) was found when pain was defined as self-reported daily pain at rest [6], but the prevalence of pain was considerably higher when assessed as bothersome pain (52%–70.8%) [9, 35, 50]. The highest reported prevalence of pain was 87.5%, which was found in a study exploring any present pain [5]. The results of any present pain in PLWD at home are in line with the findings in a systematic review of present pain among PLWD in nursing homes (NH) [21]. Thus, the overall high prevalence of pain found in this review and the review from PLWD in NH [21] shows that pain in PLWD is a symptom of clinical importance. Consequently, pain should be a substantial focus for healthcare service programs for PLWD, independent of where they are located.

As mentioned in the introduction, pain among older adults with dementia has been linked to medical conditions [25–28]. However, studies reporting prevalence of pain in PLWD at home by categories of medical conditions were lacking, but one study among PLWD explored the prevalence of pain in the context of frailty. They found that those living at home with dementia and frailty had pain more often than those without frailty living at home with dementia [11]. We may speculate that the frailty partly is reasoned in medical conditions. A study from the USA, which focused on pain and other conditions important for quality of life among PLWD at home, reported in analyses adjusted for age and gender, that Afro-Americans had lower prevalence of troublesome pain than White Americans, whereas Latin-Americans had higher prevalence of troublesome pain than White Americans [9]. This finding is supported by an NH study that explored pain among residents with dementia by ethnicity [20]. A study of pain in low and middle-income countries in Asia and Latin America utilized a crude analysis and found considerable variation in the prevalence of pain among people living at home with dementia of mild severity [12], which is also pointing toward ethnical differences in the prevalence of pain. However, more advanced analysis adjusting for demographic and physical or psychological health conditions was not performed in the above-mentioned studies [9, 12]. The findings may also be partly explained by cultural differences in the expression of pain.

The study from low and middle-income countries [12] was the only study to compare pain among older persons with mild dementia with pain among those with moderate or severe dementia. The study found a significantly lower prevalence of pain among those with moderate to severe dementia (crude and adjusted analysis) [12]. These findings were the opposite of the expected consequences of neuropathological changes that occur with increasing severity of dementia [29–31]. Even so, a visual comparison of prevalence of pain between studies suggests that people living with severe dementia [5] had any present pain more often than those with less-severe dementia [36, 61]. However, the methodology varied between the studies, which limits the strength of such comparisons.

In nine studies, pain was only reported by PLWD, including two studies that explored pain among people living at home with moderate to severe dementia [5, 12]. One may question the validity of the self-reporting of pain when participants have moderate to severe dementia. As dementia progresses, a point is reached at which the self-reporting of pain becomes unreliable, partly because declining cognitive and language abilities reduce their ability to recognize and evaluate pain, understand pain-related questions, and verbally communicate pain experiences [66]. It was found that 33% of people living at home with moderate to severe dementia were not able to answer any of the three pain assessment inventories used in a study [5]. In studies of PLWD at home where both caregivers and the PLWD responded to questions about pain, the agreement between the respondents was moderate [5, 56, 63]. The accuracy in pain assessments may be explained by the characteristics of the assessment inventory used [7]. In this review, caregivers responding about pain on behalf of PLWD answered one or a few single questions, except for one study [7] that used two observational multidimensional assessment inventories. When PLWD are reliant on caregivers or care workers to conduct assessments, it is recommended, in addition to education and training in the observation of pain, to use observational multidimensional pain assessment inventories to secure the accuracy of the findings [31, 67].

Overall, and independent of the assessments used, the prevalence of pain was found to be higher when reported by caregivers than by PLWD at home [56, 63]. Furthermore, when caregivers and care workers assessed pain by interpreting PLWDs’ behavior and signs, the caregiver most often reported a higher prevalence of pain than the in-home care worker [7].

However, it was also found that in one in four instances, caregivers did not report pain among those with mild to moderate severity who reported pain [63]. The reason for the lack of agreement may partly be caused by difficulties caregivers and care workers have to uncover pain among PLWD, due to either a lack of awareness of pain, misinterpretation of signals and movements from PLWD [31], and/or inadequate knowledge of the PLWD [5, 7]. Even so, the caregivers may be more familiar with the individual with dementia, so they may be more able to detect pain than paid care workers, and thus more often report higher prevalence of pain. Furthermore, we may speculate that the caregivers’ and care workers’ characteristics may influence their reporting of pain among PLWD [5]. For example, it has been found that caregivers with their own pain were more likely to report pain in PLWD with mild to moderate severity than the PLWD themselves [63]. We wonder whether caregivers with their own pain could be more sensitive to indicators of pain in PLWD’s experience of pain, [63] or whether they transferred their own pain to the PLWD. This is a topic that needs further research.

It has been reported that PLWD at home are less likely to receive any pain treatment compared with NH residents with dementia [10]. This may be explained by less health care resources in the community service. Furthermore, pharmacological pain treatment among PLWD at home is often insufficient, and the risk for insufficient pharmacological treatment increased in those with advanced dementia, reduced physical functioning, and/or high age [47]. This could be due to barriers to seeking and receiving follow-up pharmacological treatment from the general practitioner. Furthermore, insufficient treatment of PLWD at home may be recognized in pain not being uncovered by caregivers and/or care workers. Supporting this, a recent study reported pain relief as one of several palliative care needs among people living with early-onset Alzheimer’s disease [59]. Systematic community-based efforts to support caregivers [46] and care workers, including education and training to recognize and assess pain, contribute to the detection of pain among PLWD at home [68]. Furthermore, the development of guidelines for when and how care workers should assess pain [69], together with ensuring good continuity in community-based, person-centered in-home care, may help to overcome eventual barriers to pain assessment. This may also reduce the risk of the misinterpretation of pain indicators among PLWD [66] and contribute to appropriate pharmacological and non-pharmacological treatment when indicated.

### Strength and limitations

The main strength of this review is the systematic searching of several databases, and the examination of reference lists in the included studies to detect studies that were not detected in the systematic search. However, there are several limitations. One major limitation is the limited number of studies that used similar definitions of pain and same methodologies to assess prevalence of pain. This made it difficult to compare the results from the original studies and a meta-analysis was not feasible due to the heterogeneity in outcome definitions and ascertainment methods [70]. The pain prevalence reported was summarized by type of pain definition used, including any pain present within a defined timeframe (maximum 90 days), any pain present at the assessment day, pain of specified severity (e.g., troublesome pain), and lastly, pain interfering with the accomplishment of tasks. However, the number of studies withing each category of definition was low and questionnaires used in each category varied considerably. This would make a pooled prevalence estimate little reliable. Comparison between studies were further hampered by differences in the sample characteristics such as age and gender distribution, health condition, severity of dementia, and/or how dementia was diagnosed. Consequently, meta-analysis was not recommended [70].

An additional limitation is that a large proportion of the studies were small, had fair or poor methodological quality (60%), or often reported pain with one or a few single questions regardless of whether the questions were answered by or on behalf of PLWD. Thus, a pooled prevalence of pain based on these consumptions would not be recommended, and the result would not be valid. Further to be remarked is that, most of the studies were from North America or Europe, and none of the studies reported pain longitudinally.

The present study used JBI checklist for systematic reviews of prevalence studies [53] which review the risk of selection (item 1, 2, and 4), information (item 3), coverage (item 5 and 9) and classification (item 6, 7, and 8) bias. Given the limited number of studies published regarding pain in PLWD at home the last 25 years, we cannot rule out the possibility that researchers choose not to publish their results due to a perceived lack of interest in the community health service, contributing to publication bias.

### Clinical implications and future research

Pain is highly prevalent among PLWD at home and should therefore be considered a priority in community-based dementia care. Regular and systematic pain assessment is needed. Healthcare professionals, caregivers, and care workers should receive education and support in recognizing and assessing pain, and observational pain assessment tools should be considered when self-report is not feasible.

This review also highlights important research gaps. Future studies should use more standardized methodologies and validated assessment instruments to improve comparability. More research is needed on pain prevalence according to dementia severity, frailty, medical conditions, and cultural or ethnic background among PLWD at home. Longitudinal studies are also needed.

## Conclusion

The findings of this review indicate that pain is common and clinically important among PLWD at home. Considerable variation in prevalence estimates was observed across studies, reflecting differences in pain definitions, assessment methods, and respondents. Despite these methodological differences, the consistently high prevalence of pain highlights the importance of systematic pain assessment and pain management in PLWD. Future research should use more standardized methodologies to improve comparability across studies and strengthen the evidence base for pain assessment and management in this population.

## Supplementary Information

Supplementary Material 1.

Supplementary Material 2.

## Data Availability

All information used in this study was collected from previously published studies and is publicly available. All data generated or analyzed are included in this published article and its supplementary information files.

## Abbreviations

BPI-SF: Brief Pain Inventory - Short Form
CI: Cognitive Impairment
CS: Cross-Sectional Study
DSM-III-R: Diagnostic and Statistical Manual of Mental Disorders (3rd edition)
ESAS-r: Edmonton Symptom Assessment Questionnaire
EQ-5D: EuroQol 5 Dimensions
FAST: Functional Assessment Staging Tool
ICD-9-CM: International Classification of Diseases (9th revision), Clinical Modification
IQR: Interquartile Range
JBI: Johanna Briggs Institute
GDS: Global Deterioration Scale
GP: General Practitioner
MMSE: Mini Mental State Examination Score
NH: Nursing home
NHATS: National Aging and Trends Study
NPS: Neuropsychiatric Symptoms
PAG: Periaqueductal Gray
PAINAD: Pain Assessment in Advanced Dementia
PAINE: Pain Assessment in Non-Communicative Elderly Tool
PPI: Present Pain Indicator
PIS: Pain Intensity Scale
PLWD: People Living with Dementia
QoL: Quality of Life
Revised; DSM-IV: Diagnostics and Statistical Manual of Mental Disorders (4th edition)
RCT: Randomized Clinical Trial
SD: Standard Deviation
SM-EOLD: Symptom Management in End-of-Life Dementia Scale with One Pain Item
UK: United Kingdom
VAS: Visual Analog Scale
VDS: Verbal Descriptor Scale
