# Supplementary material for: Systematic review of prevalence of pain among people with dementia living in the community
Source: BMC Neurol. 2026 Jun 25;26:605. doi: 10.1186/s12883-026-05071-5 (PMC13599158; doi:10.1186/s12883-026-05071-5)
Supplement: Supplementary file 2 [file 12883_2026_5071_MOESM2_ESM.docx]

**Table S2: The systematic searches performed in all databases**

| Database | Search and records found |
| --- | --- |
| PubMed | **(((("people living with dementia") OR (dementia[MeSH Terms])) OR (cognitive impairment[MeSH Terms])) AND (pain[MeSH Terms]) AND (2000:2025[pdat])) AND (prevalence)**  **Filters: English**  Results*  10.09.2025 = 456 Records  03.02.2026 = 14 Records |
| AgeLine | **(((("people living with dementia") OR (dementia[MeSH Terms])) OR (cognitive impairment[MeSH Terms])) AND (pain[MeSH Terms]) AND (2000:2025[pdat])) AND (prevalence)**  **Filters: English**  Results*  11.09.2025 = 15 Records  03.02.2026 = 0 Records |
| Cinahl | **(((("people living with dementia") OR (dementia[MeSH Terms])) OR (cognitive impairment[MeSH Terms])) AND (pain[MeSH Terms]) AND (2000:2025[pdat])) AND (prevalence)**  **Filters: English**  Results*  11.09.2025 = 50 Records  03.02.2026 = 5 Records |
| PsycINFO | **(((("people living with dementia") OR (dementia[MeSH Terms])) OR (cognitive impairment[MeSH Terms])) AND (pain[MeSH Terms]) AND (2000:2025[pdat])) AND (prevalence)**  **Filters: English**  Results*  11.09.2025 = 197 Records  03.02.2026 = 5 Records |
| MedLine | **(((("people living with dementia") OR (dementia[MeSH Terms])) OR (cognitive impairment[MeSH Terms])) AND (pain[MeSH Terms]) AND (2000:2025[pdat])) AND (prevalence)**  **Filters: English**  Results*  11.09.2025 = 13 Records  03.02.2026 = 10 Records |
| Idunn | **(Smerte* OR Pain) (dementia OR cognitive impairment OR demens OR kognitiv svikt)**  Results*  11.09.2025 = 7 Records  03.02.2026 = 4 Records |
| Cochrane | **(((("people living with dementia") OR (dementia[MeSH Terms])) OR (cognitive impairment[MeSH Terms])) AND (pain[MeSH Terms]) AND (2000:2025[pdat])) AND (prevalence)**  **Filters: English**  Results*  11.09.2025 = 57 Records  03.02.2026 = 13 Records |

*Duplicates were not included
